# Ultra-Processed Food Consumption Associated with Overweight/Obesity among Chinese Adults—Results from China Health and Nutrition Survey 1997–2011

**DOI:** 10.3390/nu13082796

**Published:** 2021-08-15

**Authors:** Ming Li, Zumin Shi

**Affiliations:** 1 Centre for Population Health Research, Division of Health Sciences, University of South Australia, City East Campus, Adelaide, SA 5001, Australia; Ming.Li@unisa.edu.au; 2 Human Nutrition Department, College of Health Sciences, QU Health, Qatar University, Doha 2713, Qatar

**Keywords:** ultra-processed food, long-term consumption, overweight/obesity, adults

## Abstract

The association between the consumption of ultra-processed food (UPF) with overweight/obesity in Chinese adults has not been investigated. This study included a cohort of 12,451 adults aged >20 years who participated at least twice in the China Nutrition and Health Survey (CNHS) during 1997–2011. Food intake at each survey was assessed using a 3-day 24-h dietary recall. Body weight (kg), height (m), and waist circumference (WC) were measured during the survey. UPF was defined by the NOVA classification. Mixed effect logistic regression analyses were used. The mean UPF consumption of the study population (baseline mean age 43.7 years) increased from 12.0 g in 1997 to 41.5 g in 2011 with the corresponding proportion of UPF in daily diet from 1.0% to 3.6%. The adjusted odds ratios (95% CI) for BMI ≥ 25 kg/m^2^ for those with mean UPF consumption of 1–19 g/d, 20–49 g/d, and ≥50 g/d were 1.45 (1.26–1.65), 1.34 (1.15–1.57), and 1.45 (1.21–1.74), respectively (*p*-trend = 0.015), compared with the non-consumers. Similarly, the corresponding adjusted ORs (95% CI) for central obesity were 1.54 (1.38–1.72), 1.35 (1.19–1.54), and 1.50 (1.29–1.74). Higher long-term UPF consumption was associated with increased risk of overweight/obesity among Chinese adults.

## 1. Introduction

The world prevalence of overweight/obesity has tripled in the past four decades and reached 52% in adults aged 18 years in 2016 [1]. While in China, the burden was reached within two decades from 1993 to 2015, to the level of 41% for overweight, 15% for obesity, and 47% for abdominal obesity based on the China Health and Nutrition Survey (CHNS) [2]. Overweight/obesity has a wide spectrum of health consequence including cardiovascular diseases (CVD), diabetes, musculoskeletal disorders, and common cancers (breast, colorectal, prostate, etc.) and thus poses substantial economic burden in both developing and developed nations [3,4]. Central obesity defined by waist circumference (WC) has been shown as a better predictor for CVD than body mass index (BMI). WC has a higher relative integrated discrimination index than BMI in both men and women [5].

The sharp increasing trend of overweight/obesity is in line with the dramatic social-economic development observed in China and multidimensional levels of factors has been associated with overweight and obesity in all age groups [6,7]. For example, urbanization in China has a profound impact on food supply, food preferences, and dietary patterns [8,9]. Dietary patterns have been changing from predominantly traditional patterns of home-made food consisting of natural food material towards a modern one of increased processed food and drink packs from supermarkets [10]. Certain dietary patterns or high energy-dense foods and drinks have been associated with overweight and obesity in China [11,12,13,14], yet its association with processed food as a group has not been investigated.

NOVA, a new classification based on levels of food processing, developed by researchers at the University of Sao Paulo, Brazil, and widely adopted in world literature, categorizes foods and drinks into four groups and brings a novel perspective insight into the epidemic [14]. Ultra-processed food (UPF) is defined in NOVA as the 4th Group including products of entirely industrial formulations or made from substances extracted from foods, with minimal whole foods [14]. UPF is commonly high in energy density, sugars, salt, trans fats, as well as additives, but low in protein, micronutrients, and fibers [14]. UPF takes up more than 50% of total daily energy intake in high-income countries and its consumption is increasing rapidly in middle-income countries [15,16]. The increased UPF consumption is driven by economic development and urbanization, especially in nutrition transition countries like China [8]. In addition, food choice at the individual level, based not only on nutrients profile but also on taste, convenience, and cost, drives the increased trend [17,18]. A recent synthesis of 14 observational studies from countries in Europe and American continents has shown that UPF intake is associated with an overall 26% increased risk of obesity [19].

In China, UPF (e.g., baked goods, processed meat, etc.) consumption per capita was nearly tripled from about 62 g/d in 2002 to 174 g/d in 2016 [17]. However, the association between UPF consumption and overweight/obesity has not been quantified. We aimed to assess the long-term UPF consumption during 1997–2011 using NOVA classification and to fill the knowledge gap in Chinese adults.

## 2. Materials and Methods

### 2.1. Study Design and Sample

This is a longitudinal association study based on repeated measurements of dietary intake and overweight/obesity during 1997–2011 using CHNS data.

The CHNS study was an ongoing open household-based cohort study conducted in nine provinces in China [20]. CHNS used a multistage random-cluster sampling process to select samples in both urban and rural areas. Ten waves of dietary data collection (1989, 1991, 1993, 1997, 2000, 2004, 2006, 2009, 2011, and 2015) were completed. Participants could join or leave the survey in any round. The overall response rate was ˃60% based on the first survey in 1989 and >80% based on the previous survey year [20]. This study included 12,451 participants against the following inclusion criteria (Supplementary Materials Figure S1): aged ≥20 years; having participated in least two nutrition surveys during 1997–2011; having dietary and anthropometric measures of weight, height, and WC; having plausible energy intake (800–6000 kcal/d for men, and 600–4000 kcal/d for women). The survey was approved by the institutional review committees of the University of North Carolina (USA) and the National Institute of Nutrition and Food Safety (China). Informed consent was obtained from all participants.

### 2.2. Outcome Variable: Overweight and Obesity

During household visits at each survey, anthropometric data were collected using standardized protocol by trained health workers. Height was measured without shoes to the nearest 0.2 cm using a portable stadiometer. Weight was measured without shoes and in light clothing to the nearest 0.1 kg on a calibrated beam scale. WC was measured to the nearest 0.1 cm using a Seca tape measure (Seca North America, Chino, CA, USA) at the midpoint between the lowest rib margin and the iliac crest. BMI was calculated as weight (in kg) divided by height (m) squared. Based on WHO definitions, overweight/obesity is defined as BMI ≥25 kg/m^2^, and central obesity is defined as a WC of greater than 90 cm and 80 cm for males and females, respectively [1].

### 2.3. Exposure Variable: UPF Consumption

At each survey, individual dietary intake data were collected by a trained investigator conducting a 24-h dietary recall on each of three consecutive days [20]. At the household level, all the foods consumed during the survey period were weighed and assigned to each household member. The food data were recoded and converted to nutrient intake using the corresponding updated food composition tables [21,22,23]. Around 3000 food items in the food composition tables since 1997 were categorized into four groups based on the NOVA classification [16]. As to uncertain food item(s), the food-processing status was discussed, and consensus was reached. For example, the fruity or milky drinks were UPF if containing sweeteners, preservatives, and other additives.

### 2.4. Covariates

Sociodemographic and lifestyle factors were collected at each survey using a structured questionnaire. The following constructed variables were used as indicators of socioeconomic status: education (low: illiterate/primary school; medium: junior middle school; high: high middle school or higher), per capita annual family income (recoded into tertiles as low, medium, and high), urbanization levels (recoded into tertiles as low, medium, and high).

Smoking status was categorized as non-smokers, ex-smokers, and current smokers. Alcohol consumption was recorded as yes or no. Sleep duration was recorded as <6, 7–9, and >9 h per day using data collected since 2004. Physical activity level (metabolic equivalent of task MET) was estimated based on self-reported activities (including occupational, domestic, transportation, and leisure-time physical activity) and duration using the Compendium of Physical Activities.

Two dietary patterns (traditional pattern and modern pattern) were identified in this study population [11]. The traditional one was characterized by high intakes of rice, meat, and vegetables, while the modern pattern was highly correlated with fast food, milk, and deep-fried food [11].

Hypertension was defined as systolic blood pressure ≥140 mmHg and/or diastolic blood pressure ˃90 mmHg or having known hypertension. Diabetes was defined by plasma glucose ≥7.0 mmol/L, or HbA1c > 6.5% using fasting blood sample collected in 2009 or having known diabetes.

### 2.5. Statistical Analysis

Cumulative mean UPF intake for each participant was calculated from all the proceeding years to reduce within-individual variation and to represent long-term habitual intake. For example, if a participant participated in surveys in 1997, 2000, and 2004 with x, y, and z (in grams) for UPF, the cumulative mean UPF intakes for the corresponding years were x, (x + y)/2, and (x + y + z)/3. The mean UPF intake was categorized into four levels: non-consumers, 1–19 g/d, 20–49 g/d, ≥50 g/d. We chose this cut-off based on that the serving size in the context of Chinese food is *Liang* (50 g). Sample characteristics were presented and compared by baseline UPF intake levels using ANOVA for continuous measures or chi-square tests for categorical ones.

The longitudinal association between UPF consumption and overweight/obesity defined by both BMI and WC was assessed with mixed effect logistic regression analysis. Unadjusted and adjusted odds ratios (95% CI) of the fixed part of the mixed effect models were reported with within person variation as the random part. A set of models were used: unadjusted model; model 1 adjusted for age, sex, and energy intake; model 2 further adjusted for socioeconomic status (income, urbanization, and education), fat intake, behavioral factors (smoking, alcohol drinking, and physical activity) based on model 1; model 3 further adjusted for dietary patterns based on model 2. Sensitivity analyses were conducted based on model 3 among those who attended all six waves of the survey.

Interaction between UPF intake and other covariates (sociodemographic, lifestyle) on overweight/obesity was assessed by introducing a product term in the regression model. All the analyses were performed using STATA 17.0 (Stata Corporation, College Station). Statistical significance was considered when *p* < 0.05 (two-sided).

## 3. Results

### 3.1. Population Characteristics

Among the 12,451 participants included in this study, 53.2% entered in 1997, 19.7% in 2000, 12.0% in 2004, 5.8% in 2006, and 9.4% in 2009. At baseline, the mean age of this population was 43.7 years (SD 14.7), 48.7% were males, 37.7% resided in highly urbanized areas, and 31.1% were smokers. The mean BMI was 22.7 kg/m^2^. The prevalence of overweight and central obesity was 22.6% and 25.4%, respectively. The mean daily energy, fat, protein, and carbohydrate intakes were 2271 kcal, 68.4 g, 68.9 g, and 340.2 g, respectively.

At baseline, 9.782 (79%) reported no UPF intake, while 1386 (11%) reported daily UPF consumption ≥50 g. Compared with non-consumers, having ≥50 g/d were prevalent among males (66% vs. 46%), or those having higher education (36% vs. 20%) and higher income (47% vs. 35%), or those living in highly urbanized areas (51% vs. 34%), or smokers (43% vs. 29%) (*p* < 0.001). Higher consumers (≥50 g/d) had higher intake of energy, fat and protein but lower carbohydrates, and the prevalence of hypertension was higher (19% vs. 15%, *p* < 0.001). The modern dietary pattern score was higher in higher consumers than non-consumers (0.7 vs. −0.3, *p* < 0.001). Baseline age, physical activity level, sleep duration, and the prevalence of diabetes did not differ by levels of UPF intake at baseline (Table 1).

### 3.2. UPF Consumption and BMI Change during 1997–2011

**Figure 1:**
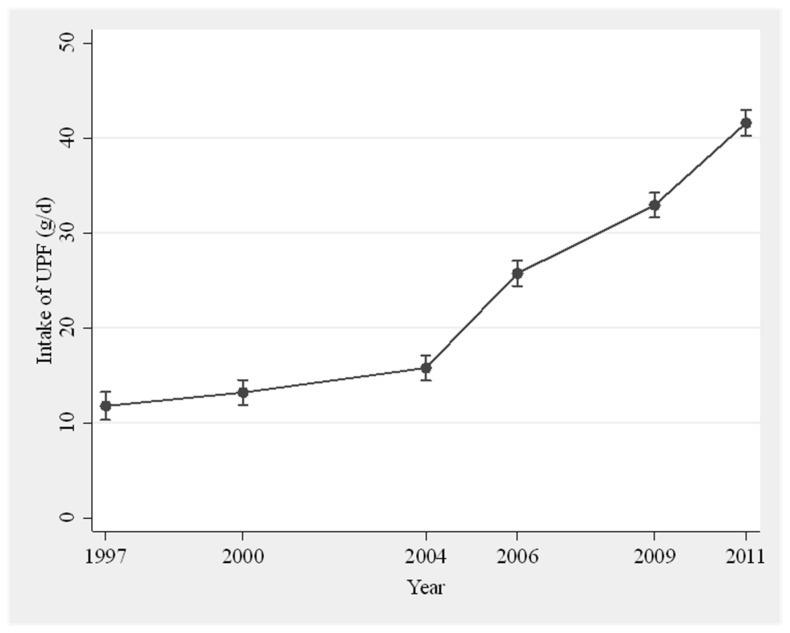
Age- and sex-adjusted mean intake of UPF in 1997–2011 (*n* = 12,451).

**Figure 2:**
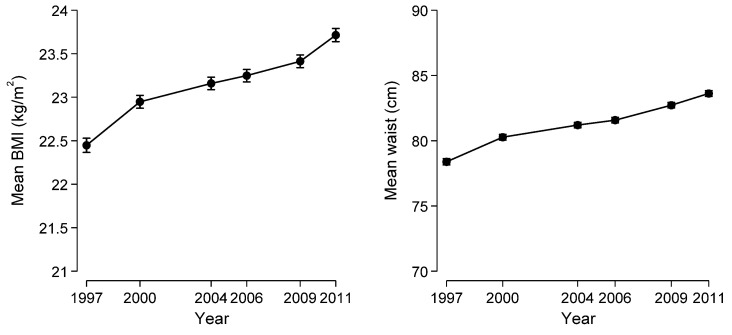
Age- and sex-adjusted mean BMI and WC in 1997–2011 (*n* = 12,451).

The top ten most frequently consumed UPF were: instant pork-mince steam bun, bread, instant noodle, cookies, cake, instant pork-mince dumpling, sausage, liquor, soybean paste, and packed snack. These foods contributed up to 51% of all UPF items. The population daily mean UPF consumption increased from 12.0 g in 1997 to 41.5 g in 2011 (Figure 1), and the proportion of UPF in daily food intake (in grams) rose from 1.0 to 3.6%. Both the intake amount and proportion climbed slowly before 2004, however, dramatically thereafter. BMI increased significantly between 1997 and 2011 with the age- and sex-adjusted mean from 22.4 to 23.7 kg/m^2^ (Figure 2). In total, 5027 participants were overweight during the period, 2816 were overweight/obese in the first wave, and 2211 developed overweight/obesity during the follow-up. Of those overweight/obese participants at baseline, 926 returned to normal weight status in some surveys. The age- and sex-adjusted mean WC increased from 78.4 cm in 1997 to 83.6 cm in 2011.

### 3.3. The Association between UPF Consumption and Overweight/Obesity

At baseline, the mean BMI of participants having UPF ≥ 50 g/d was 23.0 kg/m^2^, significantly higher than non-consumers of a mean 22.7 (*p* < 0.001, Table 1). Increased UPF consumption was significantly association with BMI ≥ 25 kg/m^2^. Compared with no UPF consumption, the unadjusted ORs for overweight/obesity were 2.35 (2.09–2.64) for 1-19 g/d, 2.18 (1.92–2.49) for 20–49 g/d, and 2.37 (2.04–2.76) for ≥50 g/d. After adjusting for age, sex, total energy intake and fat, education, income, urbanization, smoking, alcohol drinking, physical activity, and dietary patterns, the corresponding ORs were attenuated but remained significant as 1.45 (1.26–1.65), 1.34 (1.15–1.57), and 1.45 (1.21–1.74) (Table 2). Sensitivity analysis including 2231 participants participating in all six survey waves indicated that the adjusted OR (95% CI) was 1.59 (1.25–2.00) for 1–19 g/d, 1.28 (0.94–1.74) for 20–49 g/d, and 1.19 (0.80–1.79) for ≥50 g/d (Table 2).

The baseline mean WC was significantly higher in participants having UPF ≥ 50 g/d than the non-consumers (80.9 cm vs. 78.9 cm, *p* < 0.001, Table 1). Higher UPF consumption was significantly associated with central obesity defined by WC. The unadjusted ORs (95% CI) for 0–19 g/d, 20–49 g/d, and ≥50 g/d were 2.77 (2.51–3.06), 2.23 (2.00–2.50), and 2.10 (1.85–2.76) compared to no consumption. After adjusting for age, sex, total energy intake and fat, education, income, urbanization, smoking, alcohol drinking, physical activity, and dietary patterns, the corresponding ORs were attenuated but remained significant as 1.54 (1.38–1.72), 1.35 (1.19–1.54), and 1.50 (1.29–1.74) (Table 2). Sensitivity analysis including 2231 participants participating in all six survey waves indicated that the adjusted OR (95% CI) was 1.94 (1.59–2.36) for 1–19 g/d, 1.71 (1.32–2.22) for 20–49 g/d, and 1.62 (1.14–2.30) for >50 g/d (Table 2).

Other factors associated with overweight/obesity were age, education, urbanization, smoking, and physical activity. The strongest association was urbanization with the adjusted ORs (95% CI) 2.91 (2.45–3.48) for high and 1.81 (1.57–2.09) for medium compared to low urbanized areas. There was no significant interaction between UPF and these factors with overweight/obesity.

## 4. Discussion

Among a total of 12,451 participants participating in the CNHS during 1997–2011, the mean daily UPF consumption increased four times from 12.0 g in 1997 to 41.5 g in 2011 with the corresponding proportion of UPF in daily food from 1.0 to 3.6%. Higher long-term UPF consumption was associated with increased overweight/obesity by 45% and central obesity by 50%.

Among all food items classified as UPF in the Chinese food composition tables, the most frequently reported items were quite limited with the top ten taking up more than half the total items in this adult population. Those items were a combination of both popular Chinese foods such as instant pork mince stream bun and dumplings and Western foods such as bread, cookies, and sausages. The consumption pattern is different from the children and adolescent group where soft drinks, Western fast food, sweets, and chocolate are popular [13]. The per capita daily UPF consumption in China was far less than the French NutriNet-Santé cohort (4% vs. 17% of total weight) [24], although it is impossible to directly compare the amount and proportion due to different study populations, food items, and study periods. It should be noted that among a quarter of adults reported having UPF at baseline (*n* = 2669), nearly 52% (*n* = 1386) consumed more than 50 g/d.

Our data from Chinese adults confirm the evidence from most cross-sectional studies in European and American continents that higher UPF consumption was associated with overweight and obesity [19]. The strength of association of our study was 40–50%, higher than the 26% in the meta-analysis of cross-sectional studies from these countries, partly due to different study methodology. In addition, the association with central obesity defined using WC was more robust than overweight/obesity defined using BMI, indicating WC is a better estimate of visceral fat while BMI does not distinguish between the proportion of weight due to fat or muscle [5].

The positive association observed between UPFs and overweight/obesity may be partly explained by their poorer nutritional quality with higher amount of saturated fats, sugar, and energy and poorer in dietary fiber [14]. At baseline, Those having ≥50 g/d UPF had total daily energy more from fat (31%) and lower from protein (12%) than the recommended level from Chinese dietary guideline [25]. Adjusting for energy intake, and other dietary factors did not fully attenuate the association, suggesting other added non-nutritional bioactive compounds may contribute to it. Although evidence of the long-term effect on human health is lacking, some additives such as artificial sweetener, emulsifiers, thickening and stabilizing agents, and bisphenols may have impacts on body weight through pathways of insulin response, or either gut microbiota adipocyte function, or both [26]. The poor quality in nutrient profile from UPF contributes more energy [14] as a recent RCT demonstrated that an ultra-processed diet increased ad libitum energy intake by ∼500 kcal/d and caused weight gain compared with a minimally processed diet [27], and UPF was designed to boost consumption and satiate less [28]. Further research is warranted to clarify the proportional harm associated with the food physical structure and other attributes of ultra-processed foods [29].

The obesogenic environment such as heavy advertisement of UPF, large portion size, easy accessibility, and readily eating may change eating habits leading to overconsumption, less physical activity, and fast eating that can contribute to overweight/obesity [30]. We found that the age- and sex-adjusted proportion of UPF intake in daily diet increased sharply in highly urbanized areas from 1.2 to 4.2% while steadily from 0.8 to 2.1% in low urbanized areas with the gap widening since 2004 (Figure S2). Participants residing in high urbanized area tripled the risk of having overweight/obesity compared to those living in low urbanized area. This result reflected the unevenly area-based obesogenic environment transition that has been happening in China during the study period. For example, a GIS mapping of food outlets in a major city in China indicates that BMI-unhealthy outlets are four times the number of healthy outlets in the city while the density of food outlets are four times in urban area compared to the density in rural areas [31]. A nationally representative survey has indicated that environmental factors such as densities of full-service restaurants and grocery stores are synergistically associated with moderate increased risk of overweight/obesity, particularly in urban areas [32].

This is the first longitudinal study to assess the long-term UPF consumption using NOVA classification and its association with overweight/obesity in a large cohort of the Chinese adult population. The study period lasted for a relative long period of ten years. The use of mean UPF intake from three-day dietary intake in combination with household food inventory provided a robust estimate of habitual intake. The energy and food intake from the surveys are generally valid as shown from our previous investigation based on basal metabolic rate [33]. Overweight and obesity were generated from measured height, weight, and WC at each survey and classified using international definitions for cross-country comparison. We used repeated interval means for each survey to accommodate the within-person variation of UPF during the study period in the mixed effect analysis to minimize the long-term fluctuation of habitual consumption. A series of confounding factors including sociodemographic, behavioral, health, and dietary factors were adjusted.

Limitations should be noted. Firstly, misclassification was possible due to incomplete records on food processing methods in the CHNS survey not specifically designed for NOVA classification. Secondly, we used gram content to estimate the consumption of UPF which might not be precise for the diverse UPF items (e.g., soft drinks). Thirdly, due to the complexity of food processing and variabilities in additive composition between brands for a similar type of product, we could only roughly group some food items and the association could be biased. Finally, residual confounding was still possible due to the lack of data on ethnicity, which is closely related to culinary culture in China. Further well-designed studies are warranted in other populations and settings to determine causality and to identify potential mechanisms.

## 5. Conclusions

The mean daily consumption of UPF in Chinese adults increased 3.5 times from 12.0 g to 41.5 g during 2007–2011, remarkably in highly urbanized areas. Higher UPF consumption was associated with increased risk of overweight/obesity by 45–50%.

## Figures and Tables

**Table 1:** Baseline sample characteristics by levels of UPF intake (g/d): China Health and Nutrition Survey (*n* = 12,451).

Factor	None (*n* = 9782)	1–19 (*n* = 602)	20–49 (*n* = 681)	≥50 (*n* = 1386)	*p*-Value ^1^
Survey year					<0.001
1997	55.1%	63.5%	46.0%	38.4%	
2000	20.2%	14.8%	18.9%	18.4%	
2004	12.1%	10.8%	11.9%	12.0%	
2006	5.2%	4.0%	9.3%	9.1%	
2009	7.4%	7.0%	14.0%	22.1%	
Age (years), mean (SD)	43.5 (14.6)	43.7 (15.5)	43.6 (15.0)	44.6 (14.6)	0.097
Female	53.6%	55.3%	50.7%	34.1%	<0.001
Income					<0.001
Low	31.5%	24.7%	19.8%	21.4%	
Medium	33.2%	33.7%	30.8%	31.6%	
High	35.3%	41.6%	49.4%	47.0%	
Education					<0.001
Low	47.8%	43.0%	30.4%	33.3%	
Medium	32.0%	32.5%	32.0%	30.8%	
High	20.2%	24.5%	37.6%	35.9%	
Urbanization					<0.001
Low	35.8%	28.2%	19.2%	19.6%	
Medium	30.1%	26.9%	24.7%	28.9%	
High	34.1%	44.9%	56.1%	51.4%	
BMI (kg/m^2^), mean (SD)	22.7 (3.2)	22.8 (3.3)	23.2 (3.3)	23.0 (3.3)	<0.001
Waist circumference (cm), mean (SD)	78.9 (9.7)	79.3 (9.7)	80.4 (10.2)	80.9 (10.2)	<0.001
BMI >25 (kg/m^2^)	21.7%	24.1%	26.9%	26.2%	<0.001
Central obesity	24.9%	27.4%	28.2%	26.9%	0.070
Energy intake (kcal/d), mean (SD)	2250.3 (634.9)	2159.7 (598.6)	2229.7 (593.4)	2489.1 (697.6)	<0.001
Fat intake (g/d), mean (SD)	66.0 (36.0)	64.8 (34.1)	76.3 (36.1)	83.1 (40.2)	<0.001
Protein intake (g/d), mean (SD)	67.7 (22.2)	67.6 (21.4)	71.6 (22.9)	76.8 (24.8)	<0.001
Carbohydrate intake (g/d), mean (SD)	345.9 (119.9)	324.6 (114.7)	310.2 (108.8)	322.0 (112.1)	<0.001
Traditional dietary pattern score, mean (SD)	−0.0 (1.0)	0.0 (1.0)	0.1 (1.0)	0.2 (1.0)	<0.001
Modern dietary pattern score, mean (SD)	−0.3 (0.8)	−0.2 (0.8)	0.2 (1.0)	0.7 (1.2)	<0.001
Smoking					<0.001
Non-smoker	69.3%	68.8%	66.4%	53.9%	
Ex-smoker	1.3%	1.3%	2.1%	3.0%	
Current smoker	29.4%	29.8%	31.5%	43.1%	
Alcohol drinking	31.8%	34.6%	38.0%	58.2%	<0.001
Sleep duration (baseline in 2004–2009, hours)					0.81
6–9	80.7%	82.3%	77.6%	78.6%	
<6	7.8%	6.2%	9.3%	8.6%	
>9	11.5%	11.5%	13.1%	12.8%	
Physical activity (MET hours/week), mean (SD)	141.0 (117.0)	134.4 (115.4)	130.6 (112.3)	142.3 (118.9)	0.080
Diabetes (baseline in 2009 only)	9.2%	19.4%	13.4%	11.1%	0.16
Hypertension	14.7%	18.8%	15.4%	19.2%	<0.001

^1^*p* from ANOVA for continuous measures or chi-square tests for categorical ones; central obesity is defined as a WC of greater than 90 cm and 80 cm for males and females, respectively. SD is standard deviation.

**Table 2:** Association between UPF intake and overweight and central obesity among adults attending China Health and Nutrition Survey 1997–2011 (*n* = 12,451).

	None	1–19 (g/d)	20–49 (g/d)	≥50 (g/d)	*p* for Trend
**Overweight/obesity**					
Unadjusted	1.00	2.35 (2.09–2.64)	2.18 (1.92–2.49)	2.37 (2.04–2.76)	<0.001
Model 1	1.00	1.66 (1.47–1.87)	1.60 (1.39–1.83)	1.85 (1.58–2.17)	<0.001
Model 2	1.00	1.50 (1.31–1.72)	1.48 (1.27–1.72)	1.71 (1.44–2.03)	<0.001
Model 3	1.00	1.45 (1.26–1.65)	1.34 (1.15–1.57)	1.45 (1.21–1.74)	<0.001
Sensitivity analysis	1.00	1.59 (1.25–2.00)	1.28 (0.94–1.74)	1.19 (0.80–1.79)	0.070
**Central obesity**					
Unadjusted	1.00	2.77 (2.51–3.06)	2.23 (2.00–2.50)	2.10 (1.85–2.38)	<0.001
Model 1	1.00	1.84 (1.66–2.04)	1.73 (1.54–1.94)	2.04 (1.79–2.33)	<0.001
Model 2	1.00	1.63 (1.46–1.82)	1.56 (1.37–1.77)	1.90 (1.64–2.19)	<0.001
Model 3	1.00	1.54 (1.38–1.72)	1.35 (1.19–1.54)	1.50 (1.29–1.74)	<0.001
Sensitivity analysis	1.00	1.94 (1.59–2.36)	1.71 (1.32–2.22)	1.62 (1.14–2.30)	<0.001

Model 1 adjusted for age, sex, and energy intake. Model 2 further adjusted for intake of fat, income, education, urbanization, alcohol drinking, smoking, and physical activity. Model 3 further adjusted for dietary patterns [11] (traditional pattern characterized by high intake of rice, pork, and vegetables, and low intake of wheat; a modern dietary pattern had high intake of fruit, soy milk, egg, milk, and deep-fried products). Sensitivity analysis including 2231 participants of all six surveys during 1997–2011 adjusted for all variables in model 3. All the adjusted variables (except sex) are treated as time-varying covariates.

## Data Availability

The current research uses data from the China Health and Nutrition Survey (CHNS). Data described in the manuscript, code book, and analytic code are made publicly and freely available without restriction at https://www.cpc.unc.edu/projects/china (accessed on 10 May 2021).

## Supplementary Materials

The following are available online at https://www.mdpi.com/article/10.3390/nu13082796/s1, Figure S1: Sample flow chart, Figure S2: Age- and sex-adjusted proportion of UPF intake (g/d) by urbanization levels during 1997–2011 among adults attending China Health and Nutrition Survey.
