# Supplementary material for: Ultra-Processed Food Consumption Associated with Overweight/Obesity among Chinese Adults—Results from China Health and Nutrition Survey 1997–2011
Source: Nutrients. 2021 Aug 15;13(8):2796. doi: 10.3390/nu13082796 (PMC8399660; doi:10.3390/nu13082796)

Figure S1. Sample flow chart

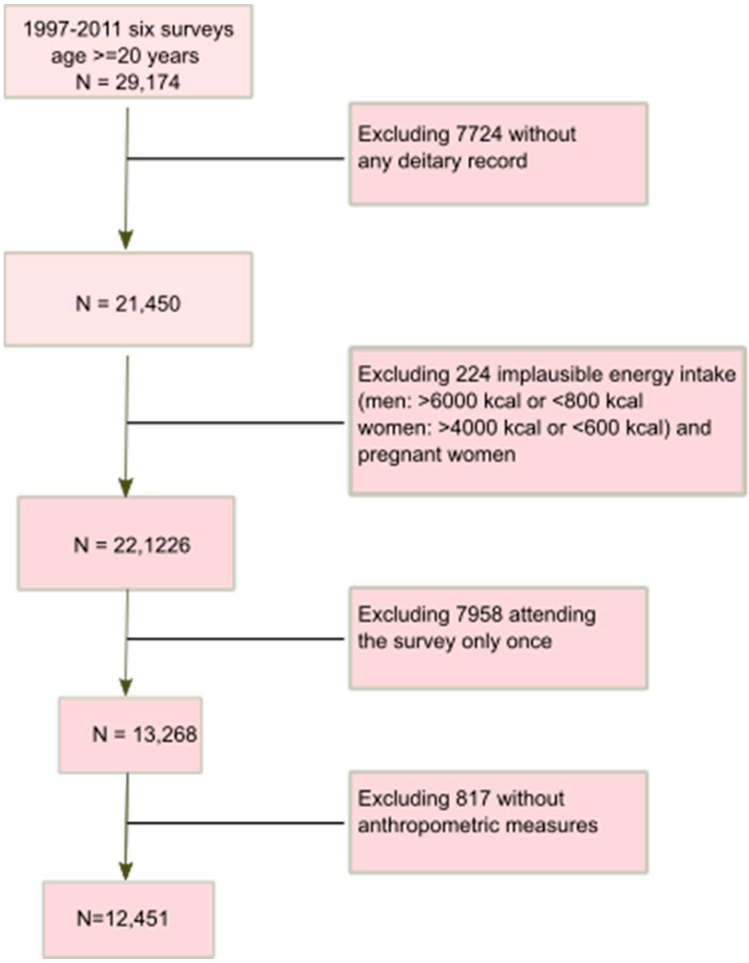

Figure S2. Age- and sex-adjusted proportion of UPF intake (g/day) by urbanization levels during 1997-2011 among adults attending China Health and Nutrition Survey

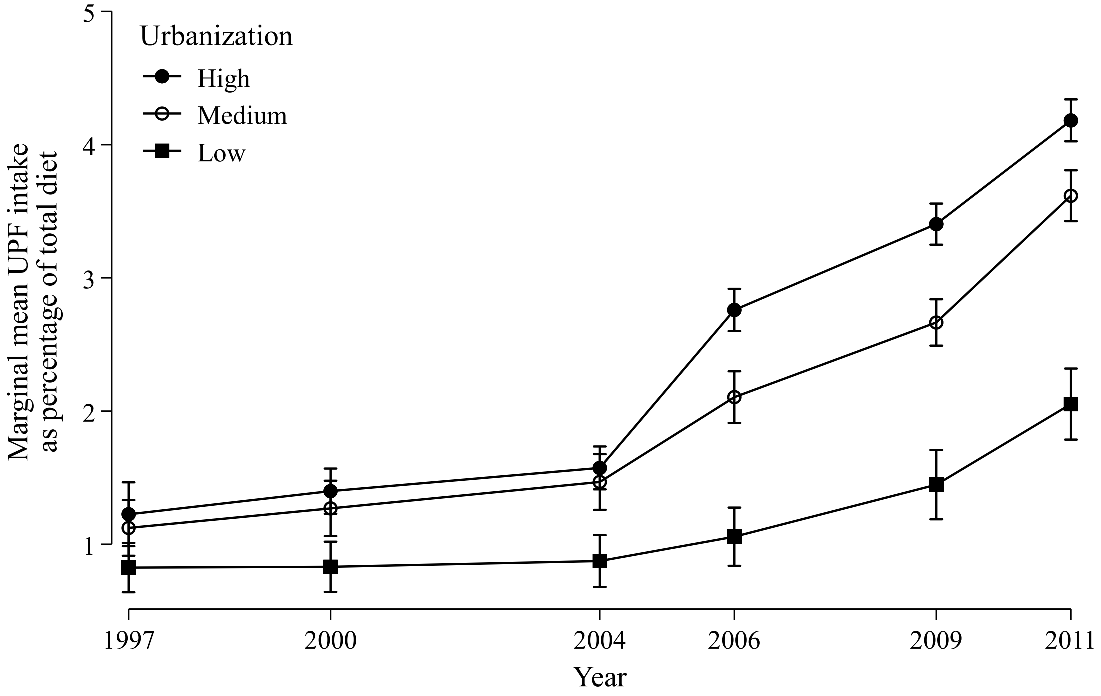

Supplement: Supplementary file 1 [file nutrients-13-02796-s001.zip › nutrients-1293311-supplementary.pdf]
